# Life course associations of height, weight, fatness, grip strength, and all‐cause mortality for high socioeconomic status Guatemalans

**DOI:** 10.1002/ajhb.23253

**Published:** 2019-05-15

**Authors:** Liina Mansukoski, William Johnson, Katherine Brooke‐Wavell, J. Andres Galvez‐Sobral, Luis Furlan, Tim J. Cole, Barry Bogin

**Affiliations:** ^1^ School of Sport, Exercise and Health Sciences Loughborough University Loughborough UK; ^2^ Centro de Investigaciones Educativas Universidad del Valle de Guatemala Guatemala Guatemala; ^3^ Centro de Estudios en Informática Aplicada Universidad del Valle de Guatemala Guatemala Guatemala; ^4^ Great Ormond Street Institute of Child Health, University College London London UK

## Abstract

**Objectives:**

The objective of this study was to investigate the association between physical growth in preadult life with five outcomes at ages 64 to 76: weight, body mass index (BMI), estimated body fat percentage, hand grip strength, and mortality.

**Methods:**

Super‐imposition by translation and rotation (SITAR) growth curves of 40 484 Guatemalan individuals aged 3 to 19 years were modeled for the parameters of size, timing and intensity (peak growth velocity, eg, cm/year) of height, weight, BMI, and grip strength. Associations between the SITAR parameters and old age outcomes were tested using linear and binary logistic regression for a follow‐up sample of high socioeconomic status (SES) Guatemalans, of whom 50 were aged 64 to 76 years at re‐measurement and 45 died prior to the year 2017.

**Results:**

SITAR models explained 69% to 98% of the variance in each outcome, with height the most precise. Individuals in the follow‐up sample who had a higher BMI before the age of 20 years had higher estimated body fat (B = 1.4 CI −0.02‐2.8) and BMI (B = 1.2, CI 0.2‐2.2) at the ages of 64 to 76 years. Those who grew slower in height but faster in weight and BMI before the age of 20 years had higher BMI and body fat later in life.

**Conclusions:**

These findings highlight the importance of a life course perspective on health and mortality risk. Childhood exposures leading to variation in preadult growth may be key to better understanding health and mortality risks in old age.

## 1 INTRODUCTION

There are two key theoretical frameworks to explain associations between early human growth and health in later life. The first is the Life course Epidemiological Model (LEM) which looks at proximate causes linking early exposures with later health. The LEM focuses on critical periods of the life course and associated risk factors, broadly defined as determinants of healthy aging (Kuh, Richards, Cooper, Hardy, & Ben‐Shlomo, 2014). This literature utilizes large cohort studies to model and identify early life predictors, later life risk factors, and disease outcomes. However, the LEM does not attempt to answer the question of why these critical periods exist in the life course in the first place, or why aging can be related back to early life events. The LEM framework is closely related to the developmental origins of health and disease (DOHaD) hypothesis, see Ben‐Shlomo, Cooper, and Kuh (2016) for a review of key similarities and differences between the models.

The second theoretical framework, called the reserve capacity hypothesis (RCH) is based on anthropological and evolutionary theory (Bogin, 2009; Crews, 2003), which aims to answer questions about the pattern of growth and aging not addressed by the LEM. Reserve capacity (RC) is defined as somatic, cognitive, social, and emotional resources that exceed the minimum required for sustaining life and allowing reproduction (Bogin, 2009; Crews, 2003). The RCH works on the assumption that building greater RC during the years of growth allows for better later‐life health. RC may be channeled into trade‐offs between greater growth, immune function, mating behavior, and/or reproduction and parental investment. In addition, the RCH predicts that individuals with greater RC at adulthood will have slower age‐related declines in physical and cognitive function.

While challenging to test empirically, both the LEM and the RCH are useful tools for understanding the possible epidemiological and evolutionary links connecting growth to later health outcomes including mortality. These frameworks are complementary and may be employed simultaneously to research early life association with later life health. A wide range of measures may be used as aging is a multifactorial process that requires a system dynamics approach (López‐Otín, Blasco, Partridge, Serrano, & Kroemer, 2013).

The aim of the present study was to model growth, and to determine associations between growth (in height, weight, body mass index [BMI], and grip strength) and later life health outcomes. This was addressed using the Universidad del Valle de Guatemala (UVG) Longitudinal Study of Child and Adolescent Development (UVG Study). The UVG Study provides the opportunity to investigate patterns of physical growth in a country with broad social, economic, and ethnic diversity, with the highest prevalence of childhood growth faltering (stunting) in Latin America, and shortest adult women in the world (NCD‐RisC, 2016), and to follow up individuals for whom reliable growth data exist. They include boys and girls between the ages of 3.6 and 20 years, from very low to very high socioeconomic status (SES) families, measured between the years 1953 and 1999. We investigate whether their growth trajectories summarized by size, timing, and intensity (defined below) in preadult life are associated with five old age outcomes––weight, BMI, estimated body fat percentage, grip strength, and mortality––in a follow‐up sample of 50 high SES participants now over 64 years old.

## 2 METHODS

### 2.1 Sample

The UVG study has been described in detail previously (Bogin, Camacho de Paz, & MacVean, 2018; Bogin & MacVean, 1983; Varela‐Silva, Bogin, Sobral, Dickinson, & Monserrat‐Revillo, 2016). The study included over 40 000 participants from seven schools in or near Guatemala City. The schools were chosen based on the SES of the students' families, as measured by the parents' education, occupation, and fees paid to the schools, and to represent ethnic diversity, primarily Ladino and Maya Guatemalans (Bogin & MacVean, 1983; Bogin, Wall, & MacVean, 1992). Maya are the indigenous population of Guatemala, while Ladinos are individuals with Spanish or mixed heritage (Bogin et al., 1992). In total, seven schools were included (one high SES, two upper‐middle SES, two lower‐middle SES and two low SES schools). All children attending the study schools over the 46‐year study period, 1953 to 1999, became participants in the study. Students were measured annually at school, during approximately the same week each year.

### 2.2 Ethics

Ethical clearance for this study was obtained from Loughborough University Ethics Approvals (human participants) Sub‐Committee and UVG Social Sciences Ethics Committee. All follow‐up participants signed an informed consent document in their native Spanish.

### 2.3 Variables

Height is a commonly used measure in human biology, easily recorded and reflective of the wider biocultural condition of society (Bogin, Silva, & Rios, 2007; Tanner, 1989). Being taller is associated with lower risk for many negative health outcomes, such as cardiovascular disease and diabetes, but higher risk for certain cancers, and positively associated with increased longevity (NCD‐RisC, 2016). Child weight, BMI (kg/m^2^) and fat mass have a more complicated relationship with later life health outcomes, but it is accepted that excessive childhood weight gain is associated with adulthood obesity, metabolic risk factors, and mortality (Araújo De Francą et al., 2016; Freedman et al., 2004; Johnson, Li, Kuh, & Hardy, 2015; Llewellyn, Simmonds, Owen, & Woolacott, 2016; Pollock, 2015).

Hand grip strength (HGS) is a commonly used indicator of overall physical ability, and low HGS is associated with frailty and mortality in older people (Rantanen et al., 1999). Systematic reviews report that low birthweight is associated with low HGS in old age and, in turn, a higher risk for dementia, disability, and mortality (Bohannon, 2008; Dodds et al., 2012). In these studies, HGS data tend to come from middle‐aged and older individuals. Less research has related childhood grip strength to old age outcomes, especially in lower‐income nations such as Guatemala. The pattern of growth in HGS, in particular its timing and intensity (peak growth velocity, for example, cm/year), has not been previously modeled.

### 2.4 Measurements

In total, anthropometric measurements of 40 484 individuals, 22 746 males and 17 738 females aged between 3.6 and 20 years were available (Table 1). The measurements followed standard protocols of the International Biological Program (Bogin & MacVean, 1978). The study data were recorded on paper forms and later digitized at the Centro de Investigaciones Educativas (Education Research Centre) UVG.

**Table 1:** Childhood sample characteristics in age and anthropometric outcomes

Variable	N	Mean (SD)
Age (years)	40 484	11.0 (3.3)
Height (cm)	40 484	138.9 (18.2)
Weight (kg)	40 479	37.2 (14.7)
BMI (kg/m^2^)	40 478	18.4 (3.2)
Grip strength (kg)	40 411	18.8 (10.7)

### 2.5 Follow‐up data collection (2017)

**Figure 1:**
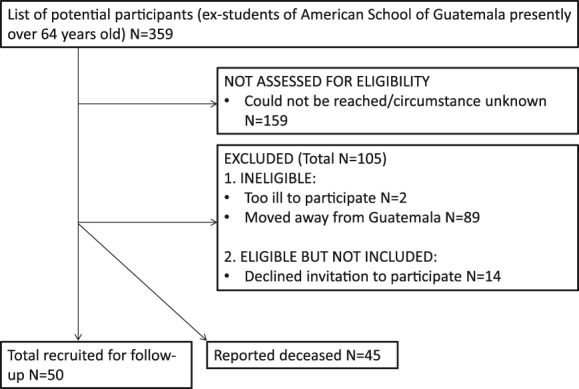
Follow‐up sample selection flowchart

During 2017, participants from the UVG study who had attended the high SES school, and were currently over 64 years old, were followed up (Figure 1). The previous contact with them was over 50 years earlier, making this follow‐up unique in its broad timespan and focus on a non‐Western population. The number of individuals was limited to those who attended the American School of Guatemala, as this school has an active alumni association and other social networks to facilitate recruitment. The American School of Guatemala is a private institution, charging relatively high fees and representing the highest parental SES of the study. Recruitment was based on direct contact, information sessions at the school, and by “snowball” dissemination of the nature of the study.

Anthropometry included height, weight, and triceps and subscapular skinfold thickness (mm) following the CDC anthropometry measuring guidelines (CDC, 2009). BMI was calculated and body fat percentage estimated from the triceps and subscapular skinfold thicknesses, following the equations of Durnin and Womersley (1973) for individuals over 50 years of age. HGS was measured in the standing position with arms by the side, using a Takei hand dynamometer. Each hand was measured twice, and the maxima averaged and recorded using the inbuilt function of the dynamometer. Participants were offered the chance to practice beforehand.

### 2.6 Data analysis

The data were analyzed in two stages. In the first stage, super‐imposition by translation and rotation (SITAR, Cole, Donaldson, & Ben‐shlomo, 2010) growth models were fitted separately for childhood height, weight, BMI, and grip strength. The 40 484 individuals contributed 157 067 measurement occasions up to the age of 20 years, a mean of 3.9 occasions per individual. The SITAR growth curve model fits a smooth mean curve to the data (as a natural cubic B‐spline), and at the same time it fits random effects for individuals that take into account differences in mean size, pubertal timing, and pubertal intensity. The random effects are each simple transformations of the mean curve that can be visualized geometrically.

*Size* indicates by how much the mean curve is shifted up or down to best match the individual's curve, where larger size is positive and smaller is negative, measured in units of the outcome (eg, cm for height).

*Timing* relates to the age at peak growth velocity (APV) and indicates by how much the mean curve is shifted left or right to best match the APV on the individual's curve, where positive timing indicates later maturation and negative indicates earlier maturation, measured in years.

*Intensity* relates to the peak growth velocity (PV), and indicates by how much the mean curve is adjusted (see below) to best match the PV on the individual's curve, where positive intensity indicates greater PV and negative indicates smaller PV, measured as a fraction of the mean PV. A child with high PV will grow “faster” than a child with a low PV (measure by eg, cm/year). The intensity adjustment can be thought of as a rotation: imagine the mean curve projected onto a half‐open door; the curve's slope changes as the door is moved, steeper on opening and shallower on closing. The slope of the curve reflects the intensity.

The key assumption of SITAR is that applying the three transformations to individual curves makes them a close match to the mean curve. Size, timing, and intensity are also estimated as fixed effects, so the random effects have mean zero (Cole et al., 2016). SITAR has been shown to provide an unbiased estimate of APV (Simpkin, Sayers, Gilthorpe, Heron, & Tilling, 2017). The SITAR formula is:

$$y_{\mathrm{it}}=\alpha_{i}+h\left(\frac{\;t-\beta_{i}}{\exp\left(-\gamma_{i}\right)}\right)$$

Where *y*_*it*_ is height for subject *i* at age *t*, *h*(*t*) is a natural cubic spline curve of height vs age, and α_*i*_, *β*_*i*_, and γ_*i*_ are subject‐specific random effects Cole et al. (2010).

To develop the most appropriate models, various transformations of the outcome and age were compared using the Bayesian Information Criterion (BIC). The model for height benefitted from a log age transformation, and the models for weight and BMI from a log transformation of the outcome. The BMI and grip strength models fitted better without the timing fixed effect. All models were fitted to girls and boys separately, as the mean curves are known to differ by sex.

In the second stage of analysis the random effects derived from the four SITAR models, expressed as Z‐scores, were used as exposures in linear regression models of the four outcomes measured at 64 to 76 years. In total, 16 models were created and the associations between each exposure––height, weight, BMI, and grip strength random effects––were compared with each outcome––weight, BMI, body fat percentage, and grip strength. All models were adjusted for sex and age at follow‐up. Birth year being collinear with age at follow‐up was not included in the models. Mortality as an outcome was related to the SITAR random effects using logistic regression. Models were run separately for males and females, as mortality trends are different (WHO, 2016). Graphs were created to visualize the growth trajectories in height, weight, BMI, and grip strength between the alive and deceased participants. Data were analyzed using SPSS (IBM, 2015) and R Statistic (R Core Team, 2018) and *P*‐value for significance was set at .05.

## 3 RESULTS

The sample recruited for follow‐up represents 14% of the N = 359 original study participants who met the selection criteria of: (a) data on physical development from at least 1 year at the American School and (b) currently aged 64 years or over (Figure 1). In total, 50 individuals agreed to participate, and 45 further individuals matching the selection criteria were reported deceased, but with no detail on cause of death. It was decided to group them as “all‐cause mortality” for analysis. See Table 2 for a summary of the four anthropometric outcome measures in the follow‐up sample.

**Table 2:** Follow‐up sample characteristics in age and anthropometric outcomes mean (SD)

	Males (N = 29)	Females (N = 21)
Age (years)	68.8 (3.5)	69.8 (3.2)
Height (cm)	172.5 (7.4)	157.4 (5.7)
Weight (kg)	78.8 (13.9)	69.5 (13.4)
BMI (kg/m^2^)	26.3 (3.5)	27.9 (4.7)
Grip strength (kg)	38.9 (5.9)	20.6 (4.0)
Body fat (%)	31.5 (4.9)	41.0 (4.9)

### 3.1 Stage 1––SITAR modeling

Ages at peak velocity and the SITAR model summaries of growth patterns in height, weight, BMI, and grip strength are presented in Table 3. The models explained 69% to 98% of the variance in the data, depending on the modeled variable. The models for height were the most accurate, with 97.8% (males) and 98.2% (females) of variance explained. Mean age at peak height velocity was 11.4 years for females and 13.3 years for males. There were modest correlations between size, timing, and intensity of growth for height, weight, and BMI (Table 3). For grip strength there was collinearity (*r* = 0.81) between size and intensity, and it was decided to model these variables both together and separately.

**Table 3:** SITAR model summaries for height, weight, BMI, and grip strength by sex^a^

Variable	SITAR model summary	Males	Females
Height (cm)^a^	N of subjects	22 746	17 738
	Median age at peak velocity	13.3	11.4
	Spline degrees of freedom	6	6
	SD of size random effect (cm)	8.0	7.1
	SD of timing random effect (%)	7.9	8.5
	SD of intensity random effect (%)	12.1	12.4
	Timing––intensity correlation	0.37	0.17
	Size––timing correlation	0.33	0.22
	Size––intensity correlation	0.64	0.48
	Variance explained (%)	97.8	98.2
Weight (kg)^a^	N of subjects	22 734	17 720
	Median age at peak velocity	13.2	11.8
	Spline degrees of freedom	5	5
	SD of size random effect (%)	15.1	14.2
	SD of timing random effect (years)	1.0	1.0
	SD of intensity random effect (%)	15.2	16.4
	Timing‐intensity correlation	−0.45	−0.21
	Size––timing correlation	−0.07	−0.02
	Size––intensity correlation	0.33	0.23
	Variance explained (%)	93.7	94.3
BMI (kg/m^b^)^a^	N of subjects	22 734	17 720
	Median age at peak velocity	14.6	12.2
	Spline degrees of freedom	4	4
	SD of size random effect (%)	12.0	12.0
	SD of intensity random effect (%)	33.2	36.2
	Size‐intensity correlation	0.45	0.33
	Variance explained (%)	84.7	84.9
Grip strength (kg)^b^	N of subjects	22 716	17 695
	Median age at peak velocity	14.4	11.8
	Spline degrees of freedom	5	4
	SD of size random effect (force kg)	3.4	3.1
	SD of intensity random effect (%)	23.9	24.8
	Size‐intensity correlation	0.81	0.81
	Variance explained (%)	77.7	69.0

a Age 3.6 to 20 years.

b Age 5 to 20 years.

### 3.2 Stage 2––Old age outcomes predicted by childhood SITAR exposures

#### 3.2.1 Weight

**Figure 2:**
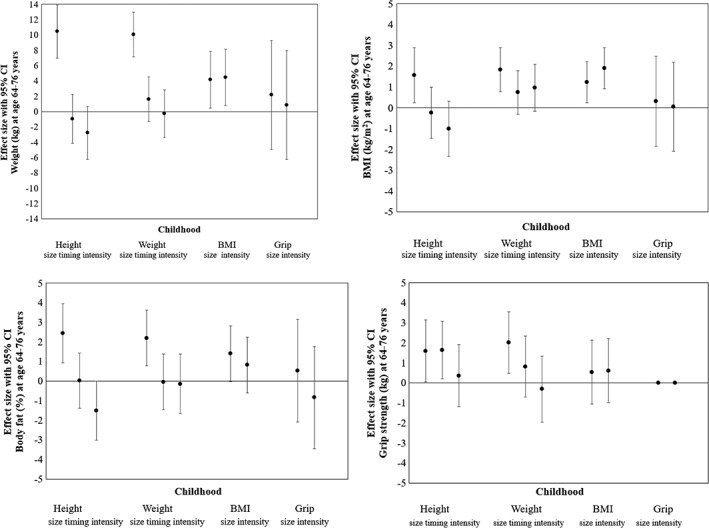
Regression coefficients for old age weight, BMI, estimated body fat (%), and grip strength as predicted by standardized childhood height, weight, BMI, and grip strength size, timing and intensity. Error bars show 95% confidence intervals. All models were adjusted by sex and age at follow‐up, N = 50

Weight increased by 4.2 kg (CI 0.5‐7.9) per SD increase in BMI size, and by 4.5 kg (CI 0.8‐8.1) per SD increase in BMI intensity (Figure 2 and Table S3). Overall, weight showed stability over the life course, whereby 61% of weight variance in old age was predicted by the pattern of weight growth.

#### 3.2.2 BMI

BMI increased by 1.6 kg/m^2^ (CI 0.3‐2.9) per SD increase in height size, by 1.8 kg/m^2^ (CI 0.8‐2.9) per SD increase in weight size, and by 1.2 kg/m^2^ (CI 0.2‐2.2) per SD increase in BMI size (Figure 2 and Tables S2 and S3). In total, 42% of BMI variance in old age was predicted by the BMI growth pattern.

#### 3.2.3 Body fat percentage

Body fat was 2.4% (CI 0.9‐4.0) higher per SD increase in height size, 2.2% (CI 0.8‐3.6) higher per SD increase in weight size, and 1.4% (0.0‐2.8) per SD increase in BMI size. Conversely, body fat was 1.5% (CI 0.0‐3.0) *lower* per SD increase in height intensity, that is, faster growth (Tables S1‐S3).

#### 3.2.4 Hand grip strength

HGS was 1.6 kg (CI 0.0‐3.1) higher per SD increase in height size, and 2.0 kg (CI 0.5‐3.5) higher per SD increase in weight size. Grip strength was also 1.6 kg (CI 0.2‐3.1) higher per SD increase in height timing, that is, later peak velocity (Tables S1 and S2). HGS size or intensity over the growth period did not predict old age HGS in this sample (Tables S4 and S5).

#### 3.2.5 Mortality

**Figure 3:**
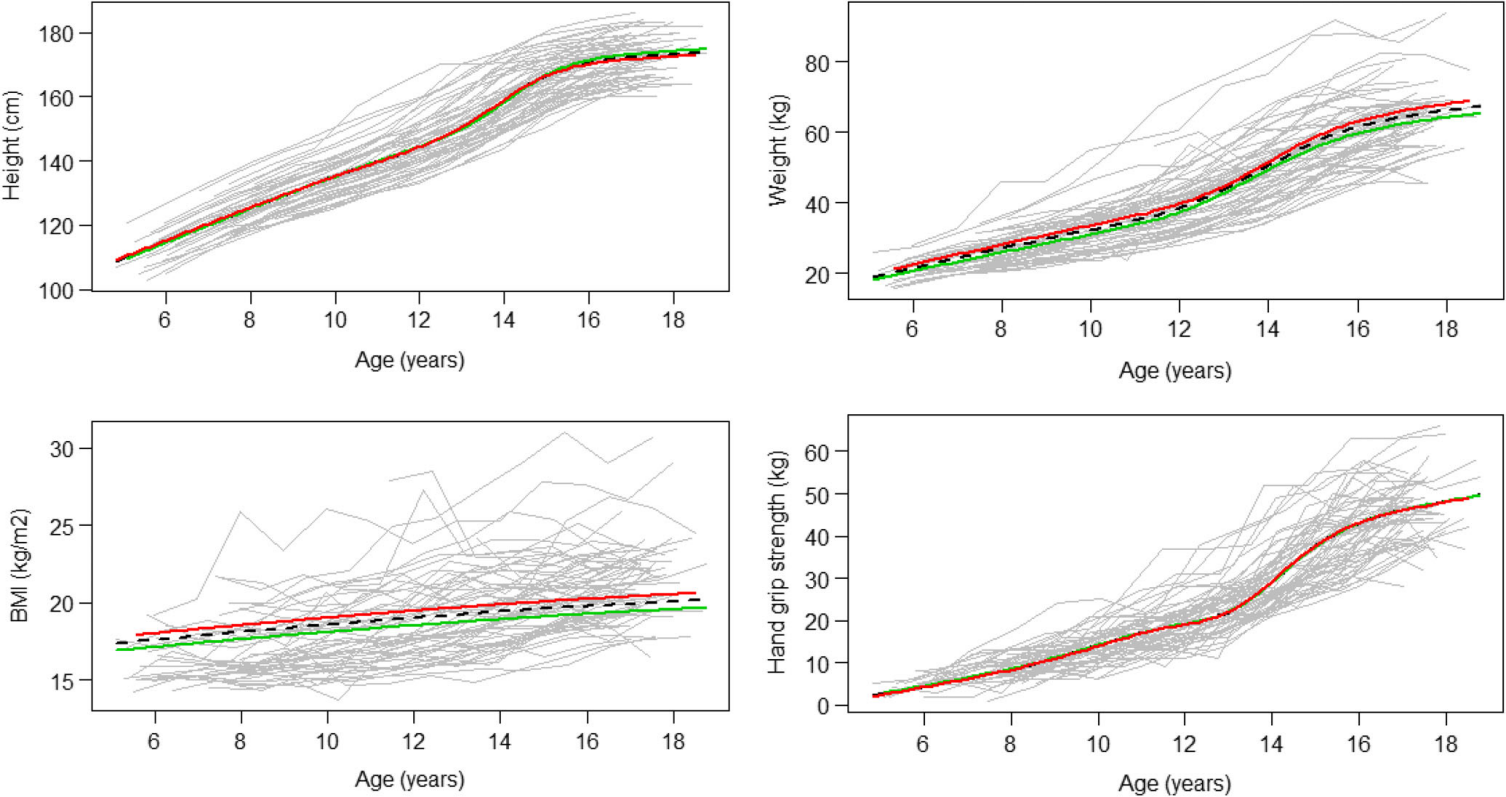
Mean male height, weight, BMI and grip strength size trajectories in the follow‐up and deceased sample (dotted line) by survivorship (green = survival to age 64 N = 29, red = deceased N = 32)

**Figure 4:**
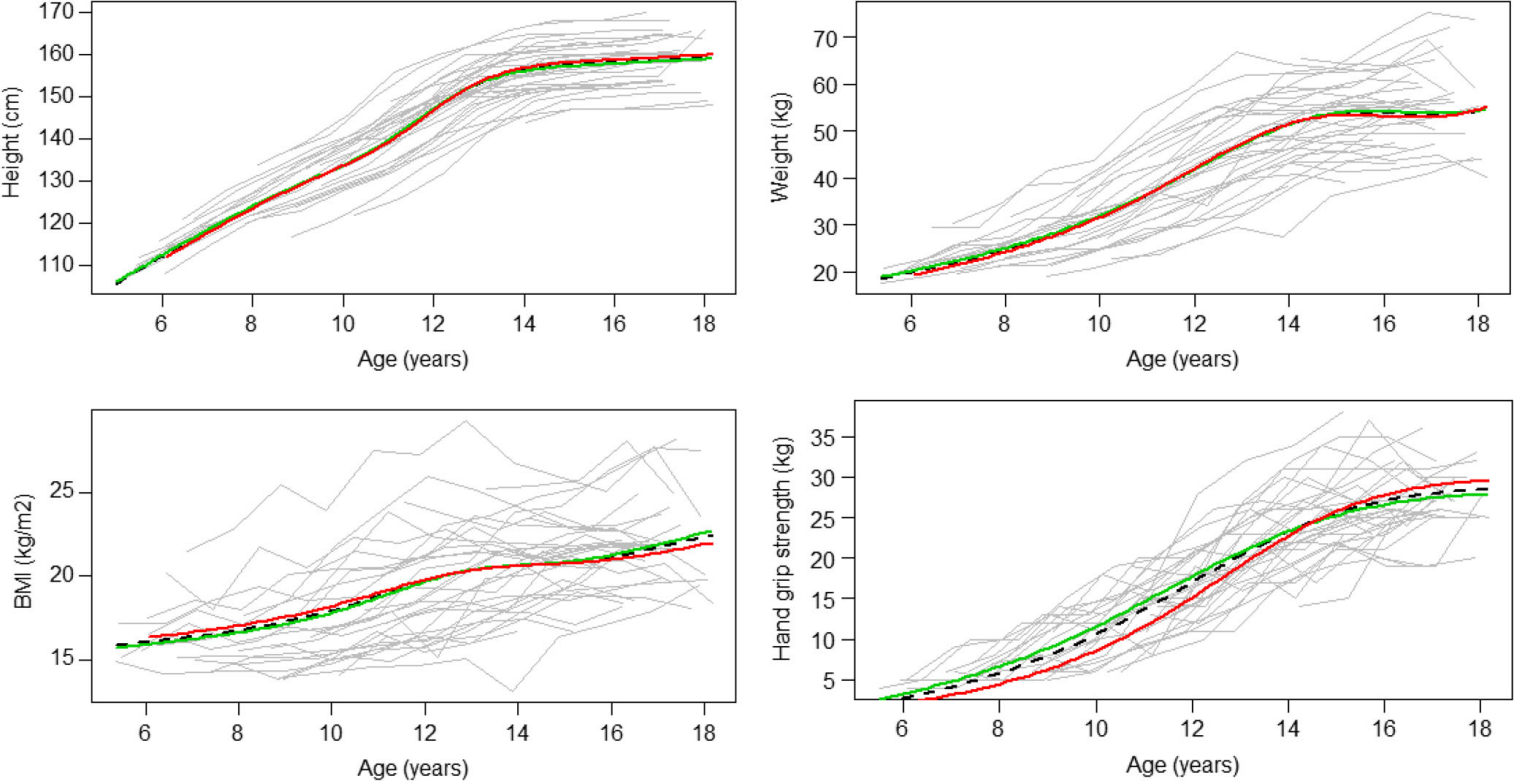
Mean female height, weight, BMI and grip strength size trajectories in the follow‐up and deceased sample (dotted line) by survivorship (green = survival to age 64 N = 21, red = deceased N = 13)

Mean trajectories for growth in height, weight, BMI, and HGS by sex between living and deceased participants are illustrated in Figures 3 and 4. The results of the logistic regression (Table 4) show that each SD increase in BMI size was associated with 42% higher odds (OR 0.58, CI 0.32‐1.04) of death prior to age 64 in males, but there was no association in females (OR: 0.9, CI 0.52‐1.90). There was a 7.65 (CI 0.92‐63.94) times higher odds of survival to age 64 for each SD increase in grip strength for women, but not men (OR 1.01, CI 0.43‐2.39) when size and intensity were included in the same model. When modeled separately, this effect was not found (Table 4).

**Table 4:** Results of logistic regression relating mortality to the patterns of growth in height, weight, BMI and grip strength; males (N = 61) and females (N = 34). Each childhood measure (height, weight, BMI, grip) was modeled separately

Childhood predictor	Males			Females		
	OR	95% CI	*P*	OR	95% CI	*P*
Height size	0.97	0.52 to 1.79	.9	0.96	0.40 to 2.27	.9
Height timing	1.37	0.75 to 2.51	.3	0.91	0.42 to 1.97	.8
Height intensity	1.62	0.87 to 3.01	.13	0.58	0.20 to 1.68	.3
Weight size	0.69	0.39 to 1.21	.2	1.25	0.62 to 2.52	.5
Weight timing	1.17	0.66 to 2.08	.6	0.79	0.37 to 1.70	.5
Weight intensity	1.05	0.60 to 1.84	.8	0.49	0.19 to 1.23	.13
BMI size	0.58	0.32 to 1.04	.07	0.99	0.52 to 1.90	.9
BMI intensity	0.97	0.56 to 1.68	.9	1.25	0.62 to 2.50	.5
Grip strength size (modeled together)	1.01	0.43 to 2.39	.987	7.65	0.92 to 63.94	.060
Grip strength intensity	1.04	0.36 to 3.01	.945	0.19	0.04 to 1.01	.051
Grip strength size (modeled separately)	1.03	0.63 to 1.70	.9	1.06	0.52 to 2.19	.8
Grip strength intensity	1.05	0.57 to 1.93	.9	0.84	0.48 to 1.46	.5

## 4 DISCUSSION

The key finding of this 50‐year follow‐up study of high SES Guatemalans is that individuals who were larger during growth––taller, heavier, and with a higher BMI, had higher BMI and body fat at 64 to 76 years. Similarly, those who grew slower in height but faster in weight and BMI before the age of 20 years had higher BMI and body fat later. Higher BMI in boys was further associated weakly with lower odds of survival to age 64. These findings are consistent with the LEM and the RCH. SITAR models summarize the whole of child growth, so it is not possible to determine whether these associations relate to specific critical periods of growth and development, for example, infancy or adolescence.

Previous research has acknowledged the need for life course epidemiology to investigate pubertal and adolescent growth spurt timing and possible related later life risks (Viner et al., 2015), but has most often focused only on absolute size (eg, height or weight) rather than the timing and intensity of growth (Cole et al., 2016). Earlier timing is linked with increased risk of adult obesity (Prentice & Viner, 2013), and children who are tall for age around puberty and with higher bone maturation are also more likely to be overweight in adulthood (Johnson et al., 2012). Early pubertal timing and higher weight gain are associated with increased risk for mental health problems, diabetes, and higher lean‐to‐fat mass ratios (Patton & Viner, 2007; Viner et al., 2015). The advantage of using SITAR modeling is that size, timing, and intensity are analyzed simultaneously and efficiently.

The general understanding in human biology is that, in most instances, “taller is better” in terms of lower risk of mortality and morbidity (NCD‐RisC, 2016). This is not supported by the present analysis. Being taller in childhood was associated with higher body fat in old age. But this was confounded in part by intensity, in that individuals whose height velocity was lower had higher body fat in old age. There is evidence that taller children are fatter, have higher BMI and leptin levels, and are more insulin resistant (Metcalf et al., 2011). There was no association between intensity of height growth and old age grip strength in our study, suggesting that the association between intensity of height growth and old age body composition may relate specifically to fat mass and not lean mass. The later life health implications of growing relatively fast or slowly are not currently known (excluding clinically significant growth pathologies, see Davies & Cheetham, 2014), nor whether nutritional and disease interventions result in noticeable changes in the intensity of growth.

Rapid weight gain during infancy is known to be associated with later obesity (Baird et al., 2005). Early timing of puberty has been reported to predict greater risk for adult obesity and higher BMI (Power, Lake, & Cole, 1997; Prentice & Viner, 2013). Based on the results of the present study, these associations may extend to the childhood, juvenile and adolescent growth stages. Faster BMI increase, and thus a more intense period of BMI growth, was positively associated with later weight, BMI and body fat.

High childhood BMI was associated with a lower odds ratio of survival to age 64 in males in the present sample, although this was not statistically significant. There was no apparent association in females, but the higher life expectancy and hence lower number of deaths in women means that this comparison was further underpowered. These observations should be explored further with a larger sample size. Odds of survival could be related to the sex difference in investments into energy storage during growing years that relate to reproductive strategies, that is, women need the early life body fat for healthy reproduction and lactation (Kuzawa, 1998; Wells, 2007).

Grip strength in old age was significantly associated with larger size during growth (both height and weight) and also later height timing. This implies, as might be expected based on allometry, that larger overall size results in more muscle mass and greater strength. Muscle strength is a key aging biomarker, and high grip strength in old age is associated with lower mortality and morbidity (Bohannon, 2008). In our study, there was an insignificant association between childhood and old age grip strength, so to clarify how growth in grip strength relates to old age grip strength further studies are needed with larger sample sizes. Finally, larger grip strength increased the odds of survival to age 64 nearly eightfold in females, if the size and intensity parameters were modeled together; however, this effect was not found when the variables were analyzed independently. This finding is due to negative confounding of the size‐survival relationship by intensity, which was positively associated with size but negatively associated with survival (see Mehio‐Sibai, Feinleib, Sibai, & Armenian, 2005).

In terms of the LEM and RCH, there are some contradictory findings. There were both positive and negative relationships of the old age outcomes with increased size or faster‐slower intensity. To better evaluate how the findings fit within the theoretical frameworks of the LEM and RCH, clarification is required on the hypothesized role of childhood weight and BMI, as specific definitions of “high” or “desirable” RC for different bodily systems and anthropometric measures may be needed.

The strengths of this study lie in the Guatemalan setting, with its differences in SES and ethnicity compared with research based in North America and Western Europe, as well as the follow‐up data collected 50 years after the original large‐scale longitudinal study. The digitized database of over 150 000 growth observations in height, weight, BMI and grip strength is an unparalleled resource for growth research, and the SITAR models built on these data are robust. Limitations relate mainly to the small size of the follow‐up sample. Recruitment for the follow‐up was very challenging as there had been no contact with the participants for nearly 50 years. There was also little available contact information, or even details of current names––many of the women had married and the name on their growth record did not match their current name. Only one researcher (LM) worked full‐time and without dedicated funding on the follow‐up recruitment. The follow‐up sample has demonstrated associations worthy of future study. Future work will require additional resources and a team of investigators to recruit a larger sample with representation from the lower SES groups and from Maya participants. These research investments are worthwhile because a life course approach is essential to understand phenotypic variation in size, body fatness, muscle performance, health, and mortality risk in old age.

## AUTHOR CONTRIBUTIONS

L.M. collected the follow‐up data, analyzed them, and prepared the first draft of the manuscript. W.J. made substantial contributions to the planning and execution of the statistical analyses and revised the manuscript. K.B.W. made substantial contributions to the design of the follow‐up data collection and revised the manuscript. J.A.G.S. and L.F. assisted with the acquisition of the follow‐up data in Guatemala City and revised the manuscript. T.C. advised on the statistical analysis using SITAR and revised the manuscript. B.B. made substantial contributions to the design of the follow‐up study, the theoretical framework underpinning the work, and revised the manuscript.

## Supporting information


**Supporting Information Table 1**. Multiple linear regressions of weight, BMI, grip strength and body fat % in old age on pattern of growth in height (standardized and mutually adjusted for size, timing and intensity), controlled by sex and age at follow‐up, N = 50.
**Supporting Information Table 2**. Multiple linear regressions of weight, BMI, grip strength and body fat % in old age on pattern of growth in weight (standardized and mutually adjusted for size, timing and intensity), controlled by sex and age at follow‐up, N = 50
**Supporting Information Table 3**. Multiple linear regressions of weight, BMI, grip strength and body fat % in old age on pattern of growth in BMI (standardized and mutually adjusted for size and intensity), controlled by sex and age at follow‐up, N = 50
**Supporting Information Table 4**. Multiple linear regressions of weight, BMI, grip strength and body fat % in old age on pattern of growth in grip strength (standardized and mutually adjusted for size and intensity), controlled by sex and age at follow‐up, N = 50
**Supporting Information Table 5**. Multiple linear regressions of weight, BMI, grip, estimated body fat % in old age on pattern growth in HGS (standardized), controlled by sex and age at follow‐up, N = 50. Size and intensity modeled separately
