# Supplementary material for: Life course associations of height, weight, fatness, grip strength, and all‐cause mortality for high socioeconomic status Guatemalans
Source: Am J Hum Biol. 2019 May 15;31(4):e23253. doi: 10.1002/ajhb.23253 (PMC6767560; doi:10.1002/ajhb.23253)
Supplement: Supplementary file 1 — Supporting Information Table 1. Multiple linear regressions of weight, BMI, grip strength and body fat % in old age on pattern of growth in height (standardized and mutually adjusted for size, timing and intensity), controlled by sex and age at follow‐up, N = 50. Supporting Information Table 2. Multiple linear regressions of weight, BMI, grip strength and body fat % in old age on pattern of growth in weight (standardized and mutually adjusted for size, timing and intensity), controlled by sex and age at follow‐up, N = 50 Supporting Information Table 3. Multiple linear regressions of weight, BMI, grip strength and body fat % in old age on pattern of growth in BMI (standardized and mutually adjusted for size and intensity), controlled by sex and age at follow‐up, N = 50 Supporting Information Table 4. Multiple linear regressions of weight, BMI, grip strength and body fat % in old age on pattern of growth in grip strength (standardized and mutually adjusted for size and intensity), controlled by sex and age at follow‐up, N = 50 Supporting Information Table 5. Multiple linear regressions of weight, BMI, grip, estimated body fat % in old age on pattern growth in HGS (standardized), controlled by sex and age at follow‐up, N = 50. Size and intensity modeled separately [file AJHB-31-na-s001.docx]

# Supporting Information

Supporting Information Table 1. Multiple linear regressions of weight, BMI, grip strength and body fat % in old age on pattern of growth in height (standardized and mutually adjusted for size, timing and intensity), controlled by sex and age at follow-up, N=50.

| **Old age outcome** | **Childhood predictor** | **B** | **95% CI**  **Lower Upper** | | **P-value** |
| --- | --- | --- | --- | --- | --- |
| Weight (kg) | Female sex | -7.8 | -14.2 | -1.3 | 0.02 |
|  | Height size | 10.5 | 7.0 | 13.9 | <0.001 |
|  | Height timing | -0.95 | -4.2 | 2.3 | 0.5 |
|  | Height intensity | -2.8 | -6.2 | 0.7 | 0.12 |
|  | Age (years) | 0.2 | -0.8 | 1.1 | 0.7 |
|  | Adjusted R^2^: 0.474 |  |  |  |  |
| BMI (kg/m^2^) | Female sex | 1.4 | -1.0 | 3.8 | 0.3 |
|  | Height size | 1.6 | 0.3 | 2.9 | 0.02 |
|  | Height timing | -0.24 | -1.5 | 1.0 | 0.7 |
|  | Height intensity | -1.0 | -2.3 | 0.3 | 0.13 |
|  | Age (years) | 0.1 | -0.3 | 0.5 | 0.6 |
|  | Adjusted R^2^: 0.071 |  |  |  |  |
| Body fat (%) | Female sex | 9.4 | 6.6 | 12.2 | <0.001 |
|  | Height size | 2.4 | 0.9 | 4.0 | 0.002 |
|  | Height timing | 0.0 | -1.4 | 1.4 | 0.9 |
|  | Height intensity | -1.5 | -3.0 | 0.0 | 0.06 |
|  | Age (years) | 0.1 | -0.3 | 0.6 | 0.5 |
|  | Adjusted R^2^: 0.555 |  |  |  |  |
| Grip strength (kg) | Female sex | -17 | -20 | -14 | <0.001 |
|  | Height size | 1.6 | 0.05 | 3.1 | 0.04 |
|  | Height timing | 1.6 | 0.2 | 3.0 | 0.03 |
|  | Height intensity | 0.4 | -1.2 | 1.9 | 0.6 |
|  | Age (years) | -0.2 | -0.6 | 0.3 | 0.5 |
|  | Adjusted R^2^: 0.805 |  |  |  |  |

Supporting Information Table 2. Multiple linear regressions of weight, BMI, grip strength and body fat % in old age on pattern of growth in weight (standardized and mutually adjusted for size, timing and intensity), controlled by sex and age at follow-up, N=50

| **Dependent Variable** | **Predictor** | **B** | **95% CI**  **Lower Upper** | | **P-value** |
| --- | --- | --- | --- | --- | --- |
| Weight (kg) | Female sex | -10.4 | -15.9 | -4.8 | <0.001 |
|  | Childhood weight size | 10.1 | 7.2 | 13.0 | <0.001 |
|  | Childhood weight timing | 1.6 | -1.3 | 4.5 | 0.3 |
|  | Childhood weight intensity | -0.3 | -3.4 | 2.9 | 0.9 |
|  | Age | 0.6 | -0.3 | 1.5 | 0.2 |
|  | R^2^: 0.612 (adjusted 0.568) |  |  |  |  |
| BMI (kg/m^2^) | Female sex | 1.3 | -0.7 | 3.3 | 0.2 |
|  | Childhood weight size | 1.8 | 0.8 | 2.9 | 0.001 |
|  | Childhood weight timing | 0.7 | -0.3 | 1.8 | 0.2 |
|  | Childhood weight intensity | 1.0 | -0.2 | 2.1 | 0.09 |
|  | Age | 0.1 | -0.3 | 0.4 | 0.5 |
|  | R^2^: 0.384 (adjusted 0.314) |  |  |  |  |
| Body fat (%) | Female sex | 9.1 | 6.4 | 11.8 | <0.001 |
|  | Childhood weight size | 2.2 | 0.8 | 3.6 | 0.003 |
|  | Childhood weight timing | -0.04 | -1.4 | 1.4 | 0.9 |
|  | Childhood weight intensity | -0.1 | -1.7 | 1.4 | 0.9 |
|  | Age | 0.2 | -0.3 | 0.6 | 0.4 |
|  | R^2^: 0.593 (adjusted 0.546) |  |  |  |  |
| Grip strength (kg) | Female sex | -18.2 | -21.1 | -15.3 | <0.001 |
|  | Childhood weight size | 2.0 | 0.5 | 3.5 | 0.011 |
|  | Childhood weight timing | 0.8 | -0.7 | 2.3 | 0.3 |
|  | Childhood weight intensity | -0.3 | -1.9 | 1.3 | 0.7 |
|  | Age | 0.02 | -0.5 | 0.5 | 0.9 |
|  | R^2^: 0.799 (adjusted 0.776) |  |  |  |  |

Supporting Information Table 3. Multiple linear regressions of weight, BMI, grip strength and body fat % in old age on pattern of growth in BMI (standardized and mutually adjusted for size and intensity), controlled by sex and age at follow-up, N=50

| **Dependent Variable** | **Predictor** | **B** | **95% CI**  **Lower Upper** | | **P-value** | |
| --- | --- | --- | --- | --- | --- | --- |
| Weight (kg) | Female sex | -12.1 | -19.4 | -4.8 | 0.002 |  |
|  | Childhood BMI size | 4.2 | 0.5 | 7.9 | 0.028 |  |
|  | Childhood BMI intensity | 4.5 | 0.8 | 8.1 | 0.018 |  |
|  | Age | 0.3 | -0.9 | 1.4 | 0.6 |  |
|  | R^2^: 0.333 (adjusted 0.274) |  |  |  |  |  |
| BMI (kg/m^2^) | Female sex | 0.7 | -1.3 | 2.6 | 0.5 |  |
|  | Childhood BMI size | 1.2 | 0.3 | 2.2 | 0.015 |  |
|  | Childhood BMI intensity | 1.9 | 0.9 | 2.9 | <0.001 |  |
|  | Age | 0.1 | -0.2 | 0.4 | 0.6 |  |
|  | R^2^: 0.420 (adjusted 0.368) |  |  |  |  |  |
| Body fat (%) | Female sex | 8.7 | 5.9 | 11.5 | <0.001 |  |
|  | Childhood BMI size | 1.4 | -0.02 | 2.8 | 0.053 |  |
|  | Childhood BMI intensity | 0.8 | -0.6 | 2.2 | 0.2 |  |
|  | Age | 0.1 | -0.3 | 0.6 | 0.5 |  |
|  | R^2^: 0.561 (adjusted 0.522) |  |  |  |  |  |
| Grip strength (kg) | Female sex | -18.5 | -21.7 | -15.4 | <0.001 |  |
|  | Childhood BMI size | 0.5 | -1.1 | 2.2 | 0.5 |  |
|  | Childhood BMI intensity | 0.6 | -1.0 | 2.2 | 0.4 |  |
|  | Age | -0.1 | -0.6 | 0.4 | 0.7 |  |
|  | R^2^: 0.765 (adjusted 0.744) |  |  |  |  |  |

Supporting Information Table 4. Multiple linear regressions of weight, BMI, grip strength and body fat % in old age on pattern of growth in grip strength (standardized and mutually adjusted for size and intensity), controlled by sex and age at follow-up, N=50

| **Dependent Variable** | **Predictor** | **B** | **95% CI**  **Lower Upper** | | **P-value** | |
| --- | --- | --- | --- | --- | --- | --- |
| Weight (kg) | Female sex | -9.4 | -17.4 | -1.3 | | 0.023 |
|  | Childhood grip strength size | 2.2 | -5.0 | 9.3 | | 0.5 |
|  | Childhood grip strength intensity | 0.9 | -6.2 | 8.0 | | 0.8 |
|  | Age | 0.5 | -0.8 | 1.8 | | 0.5 |
|  | R^2^: 0.152 (adjusted 0.076) |  |  |  | |  |
| BMI (kg/m^2^) | Female sex | 1.6 | -0.9 | 4.0 | | 0.2 |
|  | Childhood grip strength size | 0.3 | -1.8 | 2.5 | | 0.8 |
|  | Childhood grip strength intensity | 0.1 | -2.1 | 2.2 | | 0.9 |
|  | Age | 0.1 | -0.3 | 0.5 | | 0.5 |
|  | R^2^: 0.055 (adjusted -0.029) |  |  |  | |  |
| Body fat (%) | Female sex | 9.4 | 6.4 | 12.3 | | <0.001 |
|  | Childhood grip strength size | 0.5 | -2.1 | 3.1 | | 0.7 |
|  | Childhood grip strength intensity | -0.8 | -3.4 | 1.8 | | 0.5 |
|  | Age | 0.1 | -0.3 | 0.6 | | 0.5 |
|  | R^2^: 0.498 (adjusted 0.453) |  |  |  | |  |
| Grip strength (kg) | Female sex | -0.1 | -0.2 | -0.09 | | <0.001 |
|  | Childhood grip strength size | 0.003 | -0.03 | 0.03 | | 0.8 |
|  | Childhood grip strength intensity | -0.01 | -0.03 | 0.02 | | 0.7 |
|  | Age | 0.01 | 0.002 | 0.01 | | 0.011 |
|  | R^2^: 0.770 (adjusted 0.749) |  |  |  | |  |

Supporting Information Table 5. Multiple linear regressions of weight, BMI, grip, estimated body fat % in old age on pattern growth in hand grip strength (standardized), controlled by sex and age at follow-up, N=50. Size and intensity modelled separately.

| **Dependent Variable** | **Predictor** | **B** | **95% CI**  **Lower Upper** | | **P-value** | |
| --- | --- | --- | --- | --- | --- | --- |
| *Size* | | | | | | |
|  |  |  |  |  | |  |
| Weight (kg) | Female sex | -9.4 | -17.3 | -1.4 | | 0.022 |
|  | Childhood grip strength size | 2.9 | -1.03 | 6.9 | | 0.1 |
|  | Age | 0.5 | -0.8 | 1.7 | | 0.5 |
|  | R^2^: 0.150 (adjusted 0.095) |  |  |  | |  |
| BMI (kg/m^2^) | Female sex | 1.6 | -0.8 | 4.0 | | 0.2 |
|  | Childhood grip strength size | 0.4 | -0.8 | 1.6 | | 0.5 |
|  | Age | 0.1 | -0.3 | 0.5 | | 0.5 |
|  | R^2^: 0.055 (adjusted -0.007) |  |  |  | |  |
| Body fat (%) | Female sex | 9.4 | 6.4 | 12.3 | | <0.001 |
|  | Childhood grip strength size | -0.2 | -1.6 | 1.3 | | 0.8 |
|  | Age | 0.1 | -0.3 | 0.6 | | 0.5 |
|  | R^2^: 0.493 (adjusted 0.460) |  |  |  | |  |
| Grip strength (kg) | Female sex | -18.1 | -21.1 | -15.1 | | <0.001 |
|  | Childhood grip strength size | 1.1 | -0.4 | 2.7 | | 0.1 |
|  | Age | -0.04 | -0.5 | 0.5 | | 0.9 |
|  | R^2^: 0.769 (adjusted 0.754) |  |  |  | |  |
| *Intensity* | | | | | | |
| Weight (kg) | Female sex | -9.5 | -17.5 | -1.5 | | 0.021 |
|  | Childhood grip strength intensity | 2.7 | -1.3 | 6.6 | | 0.2 |
|  | Age | 0.4 | -0.9 | 1.7 | | 0.5 |
|  | R^2^: 0.144 (adjusted 0.089) |  |  |  | |  |
| BMI (kg/m^2^) | Female sex | 1.5 | -0.9 | 3.9 | | 0.2 |
|  | Childhood grip strength intensity | 0.3 | -0.9 | 1.5 | | 0.6 |
|  | Age | 0.1 | -0.3 | 0.5 | | 0.5 |
|  | R^2^: 0.053 (adjusted -0.009) |  |  |  | |  |
| Body fat (%) | Female sex | 9.4 | 6.4 | 12.3 | | <0.001 |
|  | Childhood grip strength intensity | -0.4 | -1.8 | 1.0 | | 0.6 |
|  | Age | 0.1 | -0.3 | 0.6 | | 0.5 |
|  | R^2^: 0.496 (adjusted 0.463) |  |  |  | |  |
| Grip strength (kg) | Female sex | -18.1 | -21.2 | -15.1 | | <0.001 |
|  | Childhood grip strength intensity | 1.0 | -0.5 | 2.5 | | 0.2 |
|  | Age | -0.06 | -0.5 | 0.4 | | 0.8 |
|  | R^2^: 0.767 (adjusted 0.752) |  |  |  | |  |
